# Effects of Visual Attentional Load on the Tactile Sensory Memory Indexed by Somatosensory Mismatch Negativity

**DOI:** 10.3389/fninf.2020.575078

**Published:** 2020-11-25

**Authors:** Xin He, Jian Zhang, Zhilin Zhang, Ritsu Go, Jinglong Wu, Chunlin Li, Kai Gan, Duanduan Chen

**Affiliations:** ^1^School of Life Science, Beijing Institute of Technology, Beijing, China; ^2^Intelligent Robotics Institute, School of Mechatronical Engineering, Beijing Institute of Technology, Beijing, China; ^3^Department of Psychiatry, Graduate School of Medicine, Kyoto University, Kyoto, Japan; ^4^Key Laboratory of Biomimetic Robots and Systems, Ministry of Education, Beijing, China; ^5^School of Biomedical Engineering, Capital Medical University, Beijing, China; ^6^Guangdong Country Garden School, Guangdong, China

**Keywords:** tactile sensory memory, attention, somatosensory mismatch negativity (sMMN), electroencephalogram (EEG), perceptual load theory

## Abstract

Auditory sensory memory indexed by mismatch negativity has been broadly studied over the past century, but far less attention has been directed to tactile sensory memory. To investigate whether tactile sensory memory is affected by attention, we recorded somatosensory mismatch negativity (sMMN) from 24 healthy adults in two experiments to distinguish sustained attention from non-sustained attention. Using the roving somatosensory oddball paradigm, we analyzed the average dynamic changes in the amplitude and latency of sMMN amplitude and found a clear sMMN component at the central region at a 100–300 ms interval. The sMMN amplitude, which indexes the early detection of tactile stimuli with the sensory memory trace, was larger in the tactile attentional task. Additionally, the sMMN latency increased with the increasing visual attentional load, which indicates a decay of tactile sensory memory. Our results indicate that the more attention resources are allocated for a tactile sensation, the more favorable it is to the generation of tactile sensory memory.

## Introduction

The skin covering the body’s surface contacts the external environment directly, and tactile sensations have large influences on human perception (Gallace et al., 2007). The human brain automatically encodes information from multiple tactile sensations over a short period in a real-time buffer. The tactile sensory memory described above allows us to focus on one event while still being aware of and able to process tactile events in the wider surroundings. Additionally, tactile sensory memory can be used in clinical applications (e.g., developmental coordination disorder, paralysis, and coma) to help patients improve tactile sensations or to predict the recovery of awareness. However, little attention has been paid to the topic of tactile sensory memory.

Sensory memory was originally described by Atkinson and Shiffrin (1968) using a multistore model of memory. Compared to short and long-term memory, sensory memory is an automatic and parallel preconscious response that temporarily stores incoming sensory information. This type of memory lasts only seconds and appears to show a rapidly decaying effect (Gallace and Spence, 2009). Electroencephalography (EEG) can provide high temporal information of neural components related to perception and represent various stages of information processing, and studies have used MMN to research the temporal dynamics of sensory memory (Bartha-Doering et al., 2015).

Since mismatch negativity (MMN) was first discovered by Näätänen et al. (1978), it has been suggested that MMN is generated by an automatic neural mismatch process, which consists of a memory trace that encodes the physical features of the standard stimulus (Näätänen et al., 1978, 1993; Näätänen and Michie, 1979). The MMN can be recorded when the memory trace of a repeated stimulus has not decayed (Bartha-Doering et al., 2015). Consistent with this view, previous studies showed that increasing the interstimulus offset-to-onset interval (ISI) leads to a reduction in MMN amplitude (Mantysalo and Naatanen, 1987; Bottchergandor and Ullsperger, 1992; Cowan et al., 1993; Winkler et al., 2002). So, the MMN operates at the sensory memory level.

Usually, tactile sensory memory is accessed by behavioral tasks, for example, using a sensitivity index to measure the accuracy rate on a memory set (Creelman and Macmillan, 2004; Ito et al., 2020). However, when involving in these tasks, sufficient motivation and adequate attention would be needed. So, a more objective indicator is proposed as an index of sensory memory is the MMN. Previous studies showed that the MMN is elicited irrespective of where the subject or patient’s attention is directed (Näätänen et al., 1993, 2007). Similar to the results of adult studies, prominent MMN signals can also be obtained from all waking and sleep states in infants (Cheour et al., 2000). Therefore, this pre-attentive and sensory-specific neural component provides a relatively independent relationship between attention and the sensory memory neural pathway for research.

Tactile sensory memory is considered to be outside of cognitive control; therefore, whether it is affected or modulated by attention remains unknown. Based on the comparison of sensory memory representations from preceding stimuli with that of a current deviant stimulus, some studies have proposed that MMN is unaffected by attention (Näätänen et al., 1978, 1980; Sams et al., 1984). However, Woldorff et al. (1991) carried out a dual dichotic listening experiment and found that attended tones can be distinguished from unattended tones by both ears of entry and pitch cues. They found that MMN in the unattended-channel deviant was markedly reduced compared with that in the attended-channel deviant (Woldorff et al., 1991). Subsequently, Näätänen et al. (1993) also found that the MMN intensity deviation was attenuated in the absence of attention. These studies provided the first evidence that an early sensory level in unattended channels can be attenuated or gated under highly focused attention. However, the attention of a different modality set has not been established. Other research has focused on the effects of visual attention load on auditory MMN. In that study, subjects performed a speeded letter-detection task under different attentional loads in visual modality and a simultaneous auditory oddball task. The results did not show an effect of attention on MMN. However, a follow-up meta-analysis study suggested that demanding visual tasks do reduce auditory MMN (Wiens et al., 2016). In support of these findings, recent studies found that a high visual attention load strongly reduced auditory sensory detection ability (Macdonald and Lavie, 2011; Raveh and Lavie, 2015; Szychowska et al., 2017). Moreover, somatosensory ERP studies showed that high metal workload would decrease exogenous tactile stimuli processing, but the tactile analysis was about the late positive potential component or somatosensory P2 (Sugimoto and Katayama, 2013; Mun et al., 2017). Nevertheless, other studies have reached the opposite opinion. Zhang et al. (2006) evaluated a task in which the visual attention load was parametrically manipulated by varying the number of tracked targets (Zhang et al., 2006). They found that increasing visual attention load increased auditory MMN. Overall, evidence concerning the effect of attention load on sensory memory is mixed. A large body of empirical studies over the past century has focused on the neural mechanisms associated with auditory and visual sensory memory, but few studies have focused on tactile sensory memory, particularly the effect of attention on tactile sensory memory.

In this experiment, the somatosensory mismatch negativity (sMMN) was used to measure the effects in two visual attentional tasks in case that an overlap exists between the attention pathway and the sensory memory process in a single modality (Näätänen and Gaillard, 1983). By using roving somatosensory oddball task (RSOT), which is a variant of oddball paradigm, the sMMN can be obtained by subtracting the event-related response to the standard event from the response to the deviant event (Garrido et al., 2009). To consider both sustained attention and non-sustained attention, weighting pictures and tracking balls were included in visual stimuli (Zhang et al., 2006; Debettencourt et al., 2015). And different difficulties of the visual target were used to better manipulate the process of attracting attention. The current study was aimed to investigate whether tactile sensory memory is affected by attention and analyze which pattern exists between tactile sensory memory and attention.

## Materials and Methods

### Experiment 1

#### Participants

Twelve healthy right-handed volunteers (mean age 25.3 ± 3.2 years, three females and nine males) participated in the experiment. One participant was rejected due to poor data caused by head movements. None of the participants had a history of neurological disorders or other illnesses. All the participants had either normal or corrected-to-normal vision and normal hearing. All the participants provided written informed consent before the experiment, which was approved by the ethical committee at the Beijing Institute of Technology (2017SY38).

#### Stimuli and Experiment Paradigm

**Figure 1:**
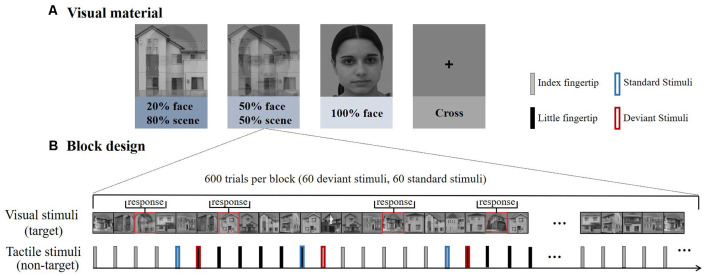
**(A)** Experiment 1 consisted of four types of blocks according to the different difficulties of visual materials. The house images were photos taken in Okayama City. The face images were obtained from the FEI face database (https://fei.edu.br/~cet/facedatabase.html). **(B)** Each block consisted of 600 trials, including 60 deviant stimuli and 60 standard stimuli. The visual stimuli and tactile stimuli were presented simultaneously.

Visual stimuli consisted of grayscale photographs of male or female faces and outdoor scenes. These images were combined into composite stimuli by averaging pixel intensities using various weightings (for example, 20% face–80% scene; Debettencourt et al., 2015). There were three types of pictures: 20% face–80% scene, 50% face–50% scene, and 100% face (Figure 1A). In addition to female faces, there were also male faces integrated with the picture, and distinguishing the gender face was a key task. Every picture was presented for 1 s. All the visual stimuli were displayed in the center of the screen at a visual angle of 10° × 10° from a viewing distance of 55 cm.

The tactile stimuli were delivered by a self-designed rigid string pressure device. The stimulus onset asynchrony (SOA) was fixed at 1 s, and the duration of each stimulus was 0.1 s. The output pressure was 1.5 N and the diameter of the rigid string was 1.5 mm. Only the rigid string part was used for delivering the stimulus to participants, the other part of the device (e.g., the pump) was placed outside the shielding room, effectively reduce the experimental irrelevant interference.

A RSOT was performed on participants in parallel with the visual stimuli. Trains of stimuli were delivered consecutively and alternatively between the subjects’ index and little fingertips of the left hand (Figure 1B). And the number of successive same-stimulus trials in a train was presented varied pseudo-randomly between four to seven. The first stimulus in each new train was modeled as “deviant,” after four to seven repetitions, the last stimulus was modeled as “standard.” So deviant and standard stimuli have the same physical properties, differing only in the number of repetitions, eliminating the interference of stimulating physical characteristics on brain responses.

#### Experiment Procedure

Participants performed the rapid gender face detection task. Whenever a target face (e.g., female face) was shown in the picture, they were instructed to press a left mouse button with their right hand as soon as possible but to ignore the tactile stimuli delivered on the left hand. To avoid any influence on tactile stimuli from pressing the button, no key tasks (female faces) appeared during the standard stimulus, deviation stimulus, and the stimulus preceding the standard stimulus. The total percentage of key task responses was 20%. In the control group, participants were instructed to fixate on the cross at the central site of the screen while counting the number of stimuli changes between their index and little fingers.

The experiment included four types of blocks (20% face, 50% face, 100% face, cross); each block consisted of 600 tactile trials (60 standard tactile stimuli and 60 deviants in each block, respectively). The first trial delivered on the index or little fingertip was randomized within each block and counterbalanced between blocks to eliminate order effects. In all the blocks, participants were seated in a chair in a sound-attenuated and electrically shielded room. Participants rested for 2 min between blocks. Within the task blocks, participants were asked to ignore the tactile stimuli and focus on the center of the visual field to attempt to detect the face stimuli as accurately as possible. They were also asked to minimize eye movements during the experiment.

### Experiment 2

#### Participants

Twelve healthy right-handed volunteers (mean age 25.1 ± 3.6, two females and 10 males) participated in the experiment. Two participants were rejected due to poor data caused by head movements. The participants had no history of neurological disorders or other illnesses. All participants had normal or corrected to normal vision and normal hearing. All the participants provided written informed consent before the experiment, which was approved by the ethical committee at the Beijing Institute of Technology (2017SY38).

#### Stimuli and Experiment Paradigm

**Figure 2:**
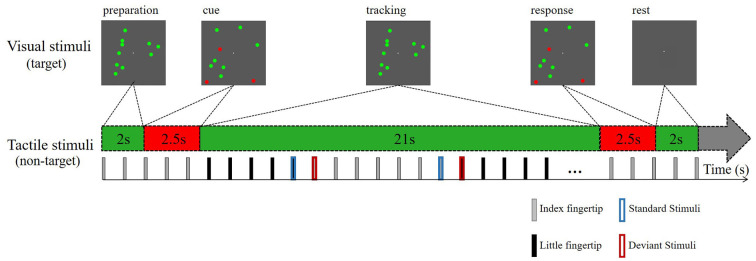
Schematic depiction of the attentive tracking task. Experiment 2 consisted of tracking three different numbers of balls during a task session lasting for 30 s. The task session was repeated twice. The participants were instructed to maintain sustained attention on the tracking tasks, ignoring the tactile stimuli.

The tactile stimuli settings were the same as those in experiment 1. For the visual stimuli, we attracted attention by presenting visual targets of varying difficulty in a visual stream. Ten bouncing balls (each 1° in diameter) moved independently at a constant velocity (2° per second) within a dark gray square (10° in both width and height). The balls moved smoothly, and there was no sudden shift in their motion. The green balls continued to move along their original path when they collided with each other but were reflected at the original speed when they impacted a screen boundary (with the reflection angle equal to the incident angle). An eye-fixation point was presented in the center of the square. Ten green balls in Brownian motion moved during the first 2.5 s of each block to engage the subject’s attention. A variable number (1, 3, or 5) of balls then turned red for 2 s and then turned back to green for the next 21 s. Therefore, the attentive tracking period lasted for 21 s (Figure 2). Then, the previously reddened balls turned red again for 2.5 s, and the participants were instructed to press the mouse button to respond whether they were tracking the right target. The entire block lasted for 30 s (2 s for rest).

#### Experiment Procedure

The participants needed to remember which ball(s) turned red, and followed ball(s) during the tracking period. And participants were instructed to press the mouse button (the left mouse button for yes, the right mouse button for no) with the right hand to respond whether they were identical to the tracked targets when the ball(s) turned red again. And they were instructed to fixate their gaze on the fixation point and to avoid eye shaking far away from the square during the attentive tracking period.

The entire experiment consisted of two task sessions and one control session (no red ball). Each task session consisted of 30 blocks (10 blocks per task condition) and lasted for 15 min. There were no differences between the two task sessions. The standard and deviant tactile stimuli were administered only during the tracking period. During the task sessions, they were asked to ignore the tactile stimuli and focus on the center of the visual field to track the balls as accurately as possible. The control session included ten green moving balls and the participants were instructed to keep their eyes on the screen but count the number of stimuli exchanges between their index and little fingers. They were also asked to minimize their eye movements during the experiment.

### EEG Recording and Processing

The subjects’ EEGs were recorded continuously (at a sampling rate of 1,000 Hz) with a SynAmps RT amplifier system using an electrode cap with 64 Ag/AgCl electrodes placed according to the 10-20 system (NeuroScan Labs, EI Paso, USA). VEOG and HEOG were recorded with two extra pairs of electrodes, one placed above and below the left eye, and the other placed on the lateral sites of eyes. The electrode impedance was kept below 5 kΩ.

Preprocessing and initial analysis of the EEG signals was performed using the EEGLAB 13.5.4b toolbox^1^ (Swartz Center for Computational Neuroscience, La Jolla, CA, USA; Delorme and Makeig, 2004) implemented in MATLAB R2014a (MathWorks Inc., Natick, MA, USA). A bandpass filter (zero phase shift, cutoff frequency 30 Hz, roll-off 12 dB/octave) was used offline on the continuous data. The reference was converted to bilateral mastoids. One and two subjects were rejected in experiment 1 and experiment 2, respectively, due to poor data caused by head movements. Independent component analysis (ICA) was used to identify and remove eye movements and other artifacts (Jung et al., 2001). Then, epochs with a duration of 1,000 ms were extracted from the continuous EEG data; each epoch extended from −200 to 800 ms relative to stimulus onset. Baseline correction was applied in a time window of 200 ms before stimulus onset. Finally, ERPs were generated separately for the index and little fingers by averaging the preprocess data epochs.

### ERP Analysis

The sMMN was calculated by subtracting the ERP waveform elicited by the standard stimuli from those elicited by the deviant stimuli. RSOT was used to avoid the difference in the physical properties between the deviant and standard stimuli. According to previous studies (Hu et al., 2013), the analysis of sMMN focused on the central scalp regions between 100 and 300 ms, and specific electrodes were selected. Point-wise paired *t*-tests were used between responses to standards and deviants in the 100–300 ms time window. For sMMN peak amplitude analysis, the negative peak in the difference wave was identified after point-wise paired *t*-tests for each participant. For sMMN latency analysis, sMMN peak latency was quantified as the latency from stimulus onset to the negative peak for each participant. And the peak amplitude and latency of sMMN were compared between conditions. In experiment 1, facial stimuli were shown to the participants, so the ERP component of N170 was extracted in a time window of 150 to 200 ms of standard stimuli to assess the degree of participants’ attention.

### Statistical Analysis

SPSS version 20.0 was used for the statistical analyses. The average sMMN waveforms of four conditions were compared. For each condition, point-wise paired *t*-tests were performed on the standard and deviant stimuli to verify that sMMN was elicited. The peak amplitudes and latency between different visual attentional conditions were tested for normality and normalized before statistical analysis and assessed *via* one-way ANOVA with Bonferroni corrections at *p* < 0.05.

## Results

### Experiment 1

#### Behavioral Data

**Figure 3:**
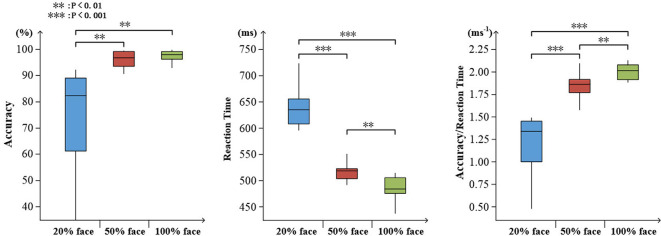
Visualized behavioral data. Statistical analysis for accuracy, reaction time, and the ratio of accuracy to reaction time (**p < 0.01; ***p < 0.001).

To determine the degree of attention, the accuracy and reaction time to the target stimuli were evaluated. As expected, an increase in task difficulty was associated with a decline in accuracy (mean ± SD: 20% face, 0.745 ± 0.200; 50% face, 0.962 ± 0.029; and 100% face, 0.977 ± 0.023) and an increase in reaction time (mean ± SD: 20% face, 637 ± 37.4; 50% face, 519 ± 26.8; and 100% face, 488 ± 21.3). There were significant effects on accuracy, reaction time and ratio of them (accuracy: *F*_(2,30)_ = 14.96, *p* < 0.001; reaction time: *F*_(2,30)_ = 141.05, *p* < 0.001; ratio: *F*_(2,30)_ = 70.48, *p* < 0.001), as illustrated in Figure 3.

#### Event-Related Potentials

##### N170

**Figure 4:**
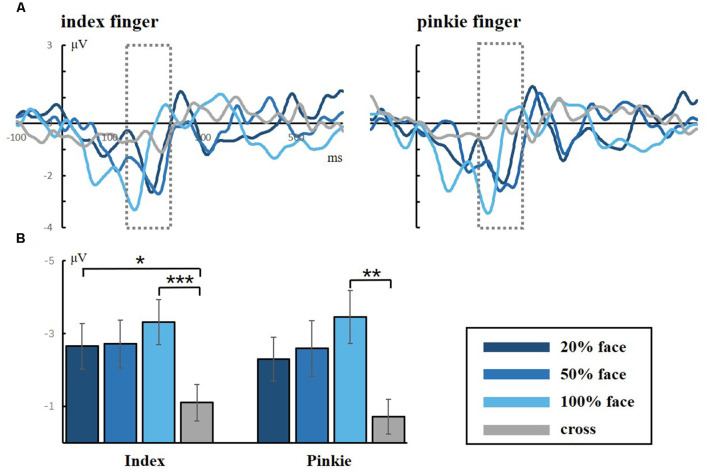
**(A)** Grand averaged responses to facial stimuli for the index and little fingers in the occipital lobe (electrode CPZ). The gray dotted lines frame the ERP component of N170, which is a facial-specific component. **(B)** Statistical analysis for the N170 peak amplitude (*p < 0.05; **p < 0.01; ***p < 0.001).

Figure 4 shows the grand averaged N170 at the electrode of CPZ. All three conditions elicited the N170 component in the standard stimuli. The one-way ANOVA for the N170 of peak amplitude showed a significant difference between the task conditions and the control condition for both the index and little fingers (index finger: *F*_(3,40)_ = 7.118, *p* = 0.001; little finger: *F*_(3,40)_ = 4.395, *p* = 0.009, respectively). Neither the index nor the little finger data showed any significant difference between task conditions, but there was an ascending tendency of N170 as the facial intensities increased. These results indicated that the visual stimuli were effective and that the attention load might increase with decreasing facial intensities.

##### sMMN

**Figure 5:**
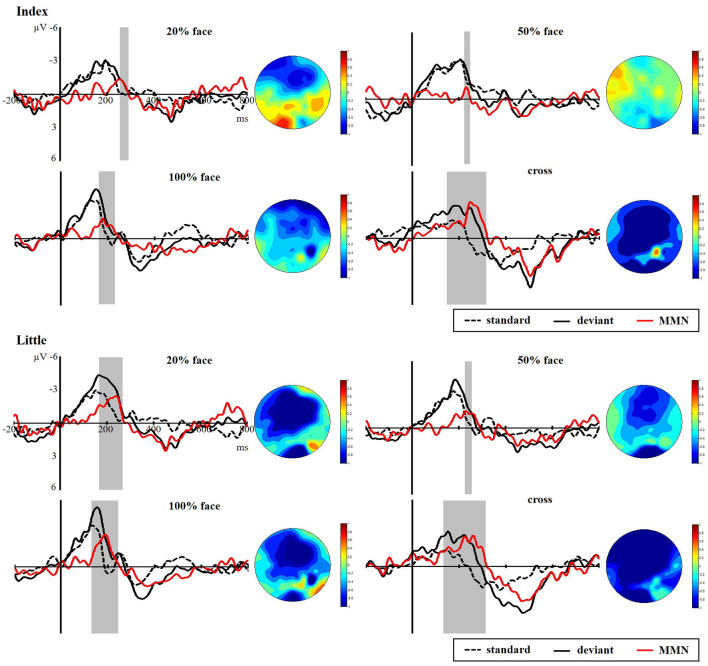
Grand average ERPs of standard (dotted line) and deviant (solid line) stimuli at the FC4 electrode site. Somatosensory mismatch negativity (sMMN; red line) obtained by subtracting the waveforms of the standard stimuli from waveforms of the deviant stimuli. The gray area superimposes on the waveform represent the time window of sMMN, showing the significant differences between the standard and deviant stimuli as revealed by the point-wise paired *t*-tests (*p* < 0.05). The scalp topographic distributions are shown beside the waveforms of ERPs for time windows of 100–300 ms.

Figure 5 presents the responses to the standard and deviant stimuli and different waves at FC4 for the index and little fingers and their scalp topographic distributions in the 100–300 ms interval. A clear negative-trending ERP response to the tactile deviant stimuli was observed approximately 100–300 ms after stimulus onset. The difference between deviant and standard stimuli was tested by paired-samples *t*-tests in the time range of 100–300 ms to verify the generation of sMMN. The sMMN was generated in both four conditions as shown in gray zones in Figure 5. As shown in the scalp topographic distributions, sMMN is also distributed at the frontal and central regions.

**Figure 6:**
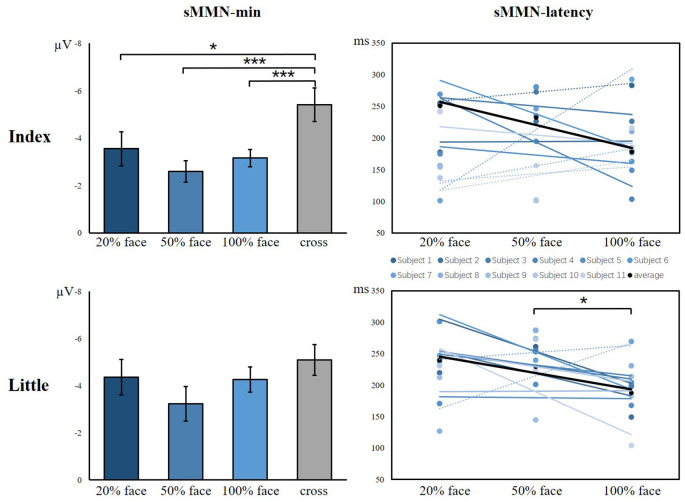
The minimum amplitude and latency of the grand averaged sMMN for the index and little fingers. The sMMN-min compares the differences between task conditions and control conditions. The sMMN latency compares differences between task conditions and the latency trend of all participants are shown (*p < 0.05; ***p < 0.001).

As shown in Figure 6, there was a significant main effect between the task conditions and the control condition, showing that the control condition elicited larger sMMN than did the task conditions in the index finger (*F*_(3,40)_ = 8.982, *p* < 0.001). Regarding the sMMN latency, there was a significant difference in the little finger (*F*_(2,30)_ = 7.169, *p* = 0.003). A Bonferroni-corrected pairwise comparison indicated significant differences in 50% face and 100% face (*p* < 0.05). Although we did not find significant latency differences for the index finger, we did find a similar tendency across the task conditions. Therefore, for both index and little fingers, there is a tendency that the sMMN latency becomes longer as the task becomes more difficult.

### Experiment 2

#### Behavioral Data

**Figure 7:**
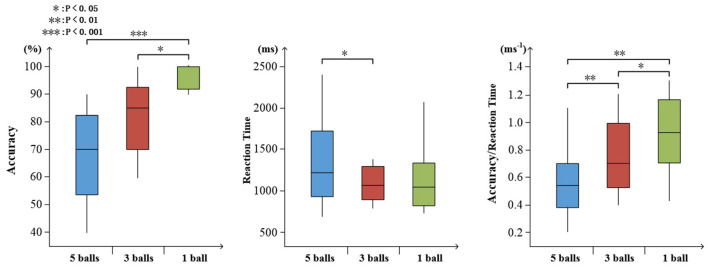
Visualized behavioral data. Statistical analysis for accuracy, reaction time, and the ratio of accuracy to reaction time (*p < 0.05; **p < 0.01; ***p < 0.001).

To determine the degree of attention, the accuracy and reaction time of target stimuli were evaluated. As shown in Figure 7, increasing task difficulty was associated with declining accuracy (mean ± SD: 5 balls 0.675 ± 0.165; 3 balls, 0.830 ± 0.132; and 1 ball, 0.973 ± 0.045) and increasing reaction time (mean ± SD: 5 balls 1.383 ± 0.507; 3 balls, 1.206 ± 0.415; and 1 ball, 1.192 ± 0.441). There were significant effects on accuracy, reaction time and ratio of them (accuracy: *F*_(2,27)_ = 15.67, *p* < 0.001; reaction time: *F*_(2,27)_ = 4.01, *p* = 0.36; ratio: *F*_(2,27)_ = 16.83, *p* < 0.001), as illustrated in Figure 7.

#### Event-Related Potentials

**Figure 8:**
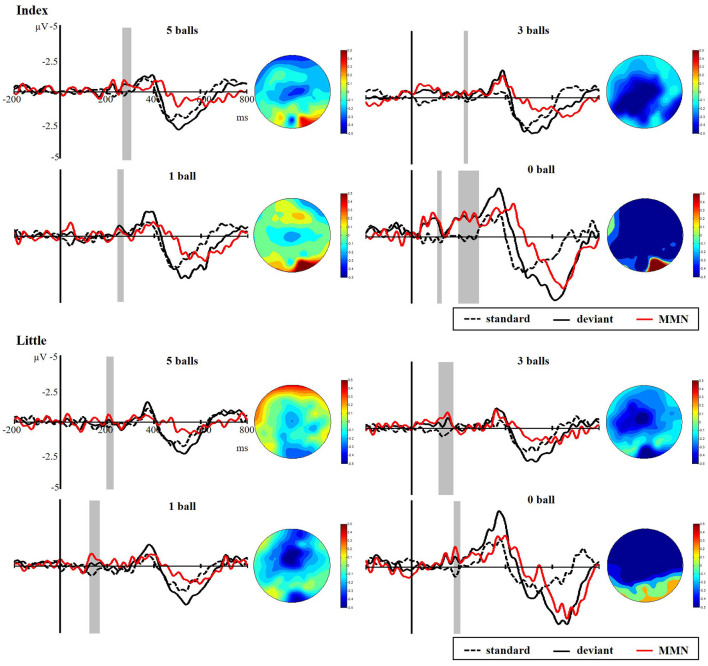
Grand average ERPs of standard (dotted line) and deviant (solid line) stimuli at the FC4 electrode site. The sMMN (red line) was obtained by subtracting the waveforms of the standard stimuli from waveforms of the deviant stimuli. The gray area superimposes on the waveform represent the time window of sMMN, showing the significant difference between standard and deviant stimuli as revealed by the point-wise paired *t*-tests. Scalp topographic distributions are shown beside the waveforms of ERPs with time windows of 100–300 ms.

Figure 8 presents the responses to standard and deviant stimuli and different waves at FC4 for index and little fingers with their scalp topographic distribution in a time window of 100 to 300 ms. The difference between deviant and standard stimuli was tested by paired-samples *t*-tests in the time range of 100–300 ms to verify the generation of sMMN. The sMMN was generated in both four conditions as shown in gray zones in Figure 8. As shown in the scalp topographic distributions, sMMN is also distributed at the frontal and central regions. Compared with experiment 1, the negative-going ERP response to the tactile deviant stimuli was smaller.

**Figure 9:**
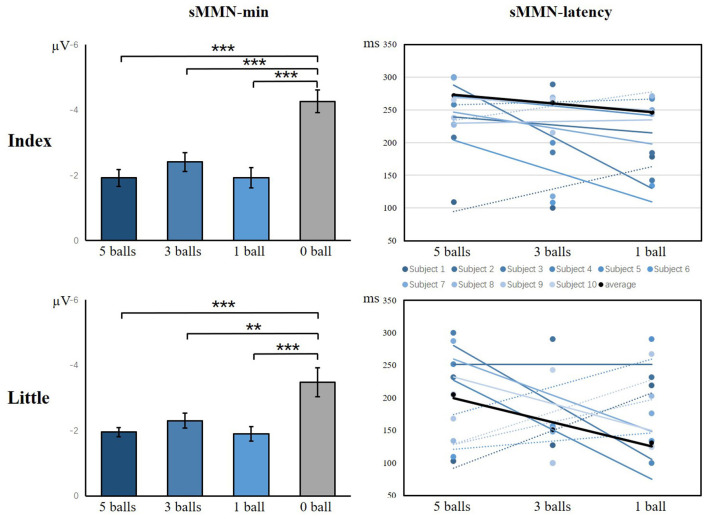
The minimum amplitude and latency of the grand averaged sMMN for the index and little fingers. The sMMN-min compares the differences between task conditions and control conditions. The sMMN latency compares differences between task conditions and the latency trend of all participants are shown (**p < 0.01; ***p < 0.001).

As illustrated in Figure 9, there was a significant main effect of the amplitude of sMMN between task conditions and control condition, showing that control condition elicited larger sMMN than any task conditions (index finger: *F*_(3,36)_ = 30.124, *p* < 0.001; little finger: *F*_(3,36)_ = 13.134, *p* < 0.001). For the latency of sMMN, neither the index nor little fingers differed between task conditions. However, they’re also a tendency that the more difficult the task was, the longer latency was, as in experiment 1.

## Discussion

This study aimed to investigate the effect of attention on tactile sensory memory. We conducted two experiments and recorded sMMN at three different difficulty levels of visual target tasks. We found that the sMMN amplitude was the largest when subjects focused on the tactile stimuli in both types of experiments but there was no significant difference based on the visual attention load tasks. However, there is an increasing latency tendency of sMMN under increasing visual task loads.

The first study of sMMN was reported that it can be elicited in the response to a change in vibration frequency (24 and 240 Hz) or spatial location (middle finger and thumb; Kekoni et al., 1997). Also, different durations of vibrotactile stimulus pairings to the fingertip can elicit an sMMN (Spackman et al., 2007). In our study, the sMMN was elicited by a change between the index and little fingertips. So, the sMMN could be stimulated by multiple stimuli of different physical characteristics, which facilitates the study of tactile sensory memory. Previous studies have investigated whether sMMN is elicited by changes to the fingers in the frontal and central regions between 100–300 ms (Hu et al., 2013; Strommer et al., 2014; Naeije et al., 2018; Zhang et al., 2019). The sMMN responses and topographies presented in this study are similar to those of previous studies. Additionally, the facial specificity component of N170 decreases as the facial intensities decrease (Bentin et al., 1996). In both sustained attention and non-sustained attention, the sMMN was elicited, tactile sensory memory can emerge in both states. But compared with experiment 1, the standard stimulus of high visual attention load in experiment 2 is nearly zero. One possible explanation is that the visual attention components continue to activate during the entire task.

Given that sMMN represents a sensory memory, we hypothesized that attention affects tactile sensory memory. Consistent with previous studies (Woldorff et al., 1991; Näätänen et al., 1993), the sMMN is significantly more negative when attention is focused completely on the tactile stimuli. This indicates that tactile sensory memory can be enhanced under a highly focused attentional state. In the non-attention task, subjects were instructed to focus on the visual targets, ignoring the tactile stimuli. That attentional resources are transferred to the visual modality to avoiding the overlapping of attention and tactile memory sensory resources in the same modality. Under different visual attentional task loads, auditory MMN may be either increased or decreased, as reported in previous studies. Here, we might have found a new and different pattern in the somatosensory system: rather than attenuating or enhancing neural responses to the task-irrelevant, the tactile sensory memory process was prolonged, and attention had no effect on their intensity. The relationship between MMN latency and pre-attentive sensory memory was reported by Tiitinen et al. (1994), who found that MMN latency could be used to predict the behavioral response latency, which was explained as originating from the pre-attentive sensory memory mechanism. MMN latency has been shown to indicate a recognition of the time of the difference between deviant and standard representations (Picton et al., 2000). Thus, reduced MMN latency may indicate a briefer involvement of the comparison process (Horton et al., 2011). And the other way around increased sMMN latency may suggest a decay of the sensory memory trace (Bartha-Doering et al., 2015). When fewer attentional resources are allocated to the tactile modality, the sMMN latency is longer, which indicates that attention may contribute to the formation of tactile sensory memory.

In both the attention and non-attention tasks, the results are broadly consistent with perceptual load theory: attention resources distributed between targets and distractors are limited (Woldorff et al., 1991; Lavie, 1995, 2010; Lavie et al., 2004). The distractors are processed less when the main task consumes all the available attention resources. Previous studies also showed that early somatosensory processing was diminished under visual load (Jones and Forster, 2013). In the present study, when subjects were instructed to count the number of stimuli exchanges between their index and little fingers during the attention task, more resources were allocated to predict errors. In this case, the sMMN amplitude, which indexes the early detection of irregular changes, would be larger. Consistently, increasing the attention load on the visual stimuli leads to increasing somatosensory memory coding time associated with detecting the incoming information regularities. It seems that our brain continues to monitor the environment, but it postpones task-irrelevant information.

Previous work showed that tactile memory can be subdivided into several functionally distinct neurocognitive subsystems, and a multi-sensory information processing network appears to play a leading role in the storage of tactile information (Gallace and Spence, 2009). Another study revealed that memory adapting properties and sensory memory capacities are presented in both the SI and SII areas, which can be considered as a model of sensory memory construction (Bradley et al., 2016). Early studies utilized a cross-modal visuo-haptic delay task to record the spikes from Brodmann’s areas 3a, 3b, 1, and 2 of monkeys and found that certain cells changed their firing frequency when they reacted to tactile objects during the presentation of a visual cue (Zhou and Fuster, 1997, 2000). The authors suggested that certain neurons are involved in responding to both tactile and visual information and that these might form part of the cross-modal memory network, which indicates that at least part of the neural network involved in the memory storage of tactile stimuli might be shared among different sensory modalities. So, some of the neurons in this cross-modal memory network might be involved in forming the generators of sMMN, and these might be the sources of multi-sensory attention that affect tactile sensory memory. However, this inference is based on previous studies, further research can operate at a neural level to investigate this assumption.

To summarize, our results demonstrate that the more attentional resources that are allocated to tactile sensation, the more favorable conditions are for generating tactile sensory memory. Here, we acknowledge some limitations: there are no significant correlations between the behavioral results and sMMN features, we will continue to further study the brain mechanism of tactile sensory memory, hoping to find better features to explain the relationship between behavioral results and brain mechanisms. Although we set up a gradient for the visual attention load and found the tendencies of N170 and sMMN in our results, a better understanding of the effects on attention and sensory memory requires further research using a better method for attracting attention. To date, the brain mechanism of tactile sensory memory has not been fully elucidated. Our research provides some evidence; however, this area still requires further exploration.

## Data Availability Statement

The raw data supporting the conclusions of this article will be made available by the authors, without undue reservation.

## Ethics Statement

The studies involving human participants were reviewed and approved by Beijing Institute of Technology (2017SY38). The patients/participants provided their written informed consent to participate in this study. Written informed consent was obtained from the individual(s) for the publication of any potentially identifiable images or data included in this article.

## Author Contributions

XH: analyzed and interpreted the data, wrote the article, and performed the experiments. JZ and ZZ: conceived and designed the experiments, and performed the experiments. CL, DC, and KG: revised the article. RG and JW: approved the final version. All authors contributed to the article and approved the submitted version.

## Conflict of Interest

The authors declare that the research was conducted in the absence of any commercial or financial relationships that could be construed as a potential conflict of interest.

## Footnotes

^1^http://sccn.ucsd.edu/eeglab/
